# Molecular Hydrogen Protects against Various Tissue Injuries from Side Effects of Anticancer Drugs by Reducing Oxidative Stress and Inflammation

**DOI:** 10.3390/biomedicines12071591

**Published:** 2024-07-17

**Authors:** Shin-ichi Hirano, Yoshiyasu Takefuji

**Affiliations:** 1 Independent Researcher, 5-8-1-207 Honson, Chigasaki 253-0042, Japan; 2 Keio University, 2-15-45 Mita, Minato-ku, Tokyo 108-8345, Japan; takefuji@keio.jp; 3 Faculty of Data Science, Musashino University, 3-3-3 Ariake, Koto-Ku, Tokyo 135-8181, Japan

**Keywords:** antitumor drug, anticancer drug, side effect, tissue injury, molecular hydrogen, reactive oxygen species, oxidative stress, inflammation, clinical application

## Abstract

While drug therapy plays a crucial role in cancer treatment, many anticancer drugs, particularly cytotoxic and molecular-targeted drugs, cause severe side effects, which often limit the dosage of these drugs. Efforts have been made to alleviate these side effects by developing derivatives, analogues, and liposome formulations of existing anticancer drugs and by combining anticancer drugs with substances that reduce side effects. However, these approaches have not been sufficiently effective in reducing side effects. Molecular hydrogen (H_2_) has shown promise in this regard. It directly reduces reactive oxygen species, which have very strong oxidative capacity, and indirectly exerts antioxidant, anti-inflammatory, and anti-apoptotic effects by regulating gene expression. Its clinical application in various diseases has been expanded worldwide. Although H_2_ has been reported to reduce the side effects of anticancer drugs in animal studies and clinical trials, the underlying molecular mechanisms remain unclear. Our comprehensive literature review revealed that H_2_ protects against tissue injuries induced by cisplatin, oxaliplatin, doxorubicin, bleomycin, and gefitinib. The underlying mechanisms involve reductions in oxidative stress and inflammation. H_2_ itself exhibits anticancer activity. Therefore, the combination of H_2_ and anticancer drugs has the potential to reduce the side effects of anticancer drugs and enhance their anticancer activities. This is an exciting prospect for future cancer treatments.

## 1. Introduction

According to statistics from the International Agency for Research on Cancer (IARC), an external research organization of the World Health Organization (WHO), there will be approximately 20 million newly registered cancer cases and 9.7 million cancer deaths worldwide in 2022 [1]. It is estimated that 1/5 of the world’s population will develop some form of cancer in their lifetime, and 1/9 of men and 1/12 of women will die from cancer; however, these figures vary from country to country and region to region [1]. Although recent advances in diagnostic and treatment technologies for cancer have improved survival rates of cancer patients, cancer remains one of the diseases that needs to be overcome. Treatment approaches to cancer are divided into three major categories: surgical, radiation, and drug therapies.

Drug therapy has a complementary or alternative role to surgery and radiation. Cytotoxic anticancer drugs, such as platinum (Pt)-based agents, anticancer antibiotics, alkylating agents, antimetabolites, topoisomerase inhibitors, and microtubule inhibitors, and molecular-targeted drugs, such as antibody drugs, and tyrosine kinase inhibitors (TKI), are often used in drug therapy [2,3]. In addition, hormone therapy drugs targeting breast and prostate cancer are also used [4,5]. Immune checkpoint inhibitors, such as anti-programmed cell death-1 (PD-1) antibody, anti-programmed cell death ligand-1 (PD-L1) antibody, and cytotoxic T-lymphocyte antigen-4 (CTLA-4) antibody, have been widely used and are called cancer immunotherapeutic drugs [6,7]. Furthermore, recent research has suggested the potential application of small molecule inhibitors targeting the RAD52 gene in breast cancer treatment, as well as the use of the tumor suppressor miR-99b-5p in prostate cancer therapy [8,9]. Extracellular vesicles, which are released from different types of cells that participate in intercellular communication to maintain physiological and pathological processes, have also been proposed as potential therapeutic candidates for the etiology of various diseases, including cancer [10].

However, the therapeutic outcomes of these anticancer drugs for cancer patients are unsatisfactory and they also cause side effects [11]. The side effects of cytotoxic anticancer drugs and molecular-targeted drugs are serious and are one of the dose-limiting factors of these drugs [12,13]. To reduce side effects, derivatives, analogues, and liposome formulations of existing anticancer drugs have been developed [12,14,15,16,17], and anticancer drugs have also been combined with antioxidant, anti-inflammatory, and anti-apoptotic substances that reduce side effects [18,19]. However, these substances have limited efficacy in reducing side effects and, thus, the emergence of substances that reduce side effects with superior efficacy and safety is desired.

In 2007, molecular hydrogen (H_2_) was reported as an antioxidant that selectively reduces hydroxyl radicals (•OH) and peroxynitrite (ONOO^−^), the most oxidizing reactive oxygen species (ROS) and reactive nitrogen species (RNS), respectively [20]. Biomedical research on H_2_ has since been conducted worldwide, with more than 2000 studies reporting the effects of H_2_ [21]. On the other hand, due to the extremely slow reaction rate of H_2_ and •OH in aqueous solution, the in vivo reaction of •OH and H_2_ remains unclear. In 2023, an iron-porphyrin oxide was shown to catalyze the reaction between H_2_ and •OH in vivo [22]. The oxide of iron-porphyrin was identified as the target molecule of H_2_; however, research on the target molecule of H_2_ is still in its early stages. H_2_ not only exerts antioxidant effects by directly reducing •OH and ONOO^−^, but also exerts indirect antioxidant, anti-inflammatory, and anti-apoptotic effects by regulating gene expression [23,24,25,26]. Although these indirect mechanisms of H_2_ are gradually being elucidated, further research is warranted. Therefore, future mechanistic studies of the efficacy of H_2_, including its target molecules, are needed.

Animal and clinical studies showed that H_2_ itself exhibits anticancer activity, and its combination with anticancer drugs achieved excellent anticancer activity [27,28,29,30]. Moreover, H_2_ was found to reduce various side effects of anticancer drugs in animal studies and clinical trials [31,32,33,34,35,36,37,38,39,40,41,42,43,44,45,46]. However, to the best of our knowledge, clinical trials have yet to focus on reductions in the side effects by H_2_ or comprehensively review these effects by H_2_ and the underlying molecular mechanisms. Therefore, we hypothesized that the antioxidant and anti-inflammatory effects of H_2_ may be involved in reducing the side effects of anticancer drugs and conducted a comprehensive literature review. We reviewed the findings of animal studies in which H_2_ protected against various tissue injuries induced by anticancer drugs, investigated the underlying molecular mechanisms, and discussed the potential clinical application of H_2_ as a substance to reduce the side effects of anticancer drugs.

## 2. Methodology

**Figure 1:**
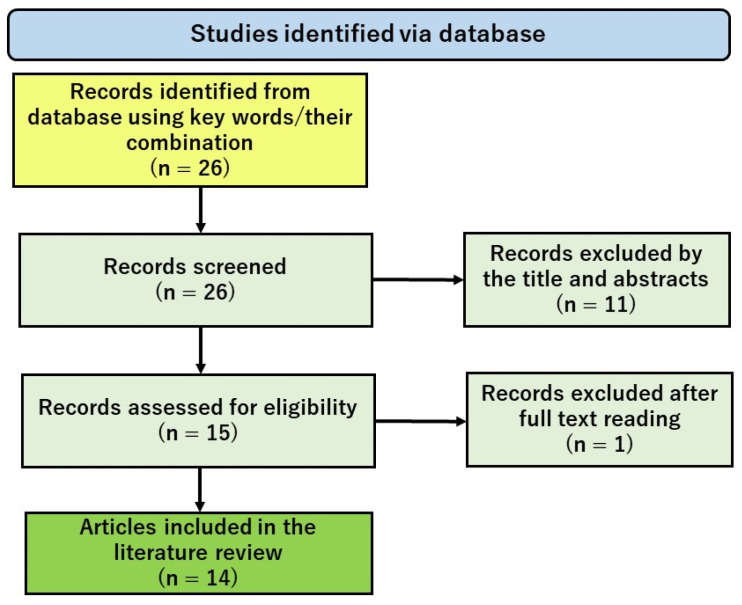
PRISMA flowchart showing the literature search and selection steps.

PubMed, a complimentary database, primarily comprises the MEDLINE database, which contains references and abstracts on life sciences and biomedical topics. The United States National Library of Medicine (NLM), part of the National Institutes of Health (NIH), maintains it as a component of the Entrez system for information retrieval. To investigate the effects of H_2_ in reducing the side effects of anticancer drugs, we performed a literature search using the PubMed electronic database on 10 January 2024. The search used Medical Subject Headings (MeSH) terms and combined keywords using the Boolean operators “AND” and “OR”. In this literature search, our preliminary search using the keywords “molecular hydrogen” AND “anticancer drug” AND “animal experiments” yielded over several thousand references. A closer look at the results of this search revealed that there was a great deal of “noise” in the search results. Since the literature we are interested in is the literature of animal studies or clinical studies showing the efficacy of H_2_ in various dosage forms against tissue injury as a side effect of anticancer drugs, we considered the following keywords and their combinations as countermeasures to eliminate the “noise” in this search. The keywords used in the search were (“molecular hydrogen” OR “hydrogen gas” OR “hydrogen water” OR “hydrogen inhalation” OR “hydrogen-rich”) AND (“cisplatin” OR “doxorubicin” OR “gefitinib” OR “bleomycin” OR “anti-tumor drug” OR “anti-tumor agent” OR “anti-cancer drug” OR “anti-cancer agent” OR “chemotherapy-induced”). The PRISMA flowchart in Figure 1 shows how to select information that is already publicly available, following the instructions described by Page et al. [47,48]. This literature search identified 14 animal studies on the Pt agents cisplatin (CIS) and oxaliplatin (OXA), the topoisomerase II (Top2) inhibitor doxorubicin (DXR), the anticancer antibiotic bleomycin (BLM), and the TKI gefitinib (GEF) (Figure 1).

## 3. Pharmacological Effects and Side Effects of Anticancer Drugs

### 3.1. CIS and OXA

CIS and OXA are Pt drugs, and both drugs share a common chemical structure with Pt drugs: a central Pt surrounded by carrier ligands and leaving groups [49]. CIS is widely used in the treatment of ovarian, head and neck, breast, uterine, gastric, and lung cancers, osteosarcoma, and malignant lymphomas [50,51]. OXA is used to treat colon, rectal, pancreatic, stomach, and small intestine cancers [52]. The carrier ligands in both drugs are tightly bound to Pt, whereas the leaving groups are weakly bound to Pt and dissociate depending on the surrounding environment, covalently binding to the purine base of the DNA strand inside the cell [49]. Although the mode of covalent binding differs between CIS and OXA, the Pt-DNA adducts formed inhibit DNA replication and transcription, leading to apoptosis [49]. CIS induces strong nephrotoxicity, which has been implicated in the ROS-mediated impairment of mitochondrial function [51]. CIS-induced vomiting and hearing impairment are also problematic. On the other hand, OXA has a higher frequency of peripheral neuropathy than other Pt drugs [52].

### 3.2. DXR

DXR is an anthracycline anticancer antibiotic isolated from a secondary metabolite of microorganisms and is widely used in the treatment of lung, breast, bladder, and uterine cancers, osteosarcoma, and malignant lymphoma [53]. DXR inhibits Top2, which is primarily involved in the cleavage and binding of double-stranded DNA, thereby inhibiting DNA replication and inducing apoptosis in cancer cells [54]. DXR causes bone marrow damage, liver dysfunction, cardiotoxicity, and gastrointestinal disorders [55]. Cardiotoxicity is dependent on the dose of DXR and occurs at a higher incidence when the cumulative dose exceeds 500 mg/m^2^ [55]. The mechanism of DXR-induced cardiotoxicity is not only that the quinone group of the aglycon in the chemical structure of DXR acts as an electron acceptor to produce ROS, but also that DXR binds directly to iron, and this complex produces ROS [56,57]. Furthermore, a mechanism by which DXR inhibits Top2β, an isozyme of Top2 that is only present in cardiomyocytes, not cancer cells, has been reported [58].

### 3.3. BLM

BLM is an anticancer antibiotic isolated from a secondary metabolite of microorganisms and is used to treat skin, head and neck, lung, uterine, thyroid, and esophageal cancers [59]. Similar to DXR, BLM forms a complex with iron [60]. BLM causes relatively mild bone marrow damage and also induces lung and skin damage [61]. BLM-induced lung damage, primarily interstitial pneumonia and pulmonary fibrosis is severe and dependent on the dose administered, with an increased risk at cumulative doses ≥ 400 mg/m^2^ [61]. While lung damage remains reversible in minor cases, the pathogenesis of clearly formed pulmonary fibrosis is progressive and responds poorly to steroid therapy [61]. The mechanisms underlying BLM-induced lung damage include the induction of apoptosis through the release of inflammatory cytokines via ROS as an acute change and chronic changes through collagen overproduction [62,63,64].

### 3.4. GEF

GEF is a molecularly targeted inhibitor of the epidermal growth factor receptor (EGFR), which selectively targets non-small cell lung cancer when administered orally [65]. GEF belongs to a class of EGFR-TKI that selectively bind to the intracellular EGFR tyrosine kinase and competitively inhibit ATP binding [65]. GEF causes acute lung injury, interstitial pneumonia, liver dysfunction, and skin rash as side effects [66]. Regarding the molecular mechanisms underlying GEF-induced lung injury, GEF acts on macrophages to activate NLR family pyrin domain containing 3 (NLRP3) inflammasomes, causing the release of interleukin (IL)-1β, and also induces the release of high-mobility group box 1 (HMGB1) by activating the inflammation-inducing molecule poly (ADP-ribose) polymerase 1 (PARP-1) [67].

## 4. Development of Therapies and Therapeutic Substances for the Side Effects of Anticancer Drugs

Many therapies and therapeutic substances have been developed to reduce the nephrotoxicity of CIS and the cardiotoxicity of DXR. However, although therapies exist to reduce BLM and GEF lung injury, very few new therapeutic agents have been developed to reduce the side effects of these anticancer drugs. Therefore, we have limited our discussion in this chapter to the development of therapies and therapeutic substances to reduce the nephrotoxicity of CIS and the cardiotoxicity of DXR.

### 4.1. Nephrotoxicity of CIS

Since the nephrotoxicity of CIS is mainly due to tubular damage, the conventional treatment for CIS has been to alleviate this damage by massive rehydration [68]. Although massive rehydration is a clinically common method of treatment to promote the elimination of CIS from the body and reduce the burden on the kidneys, it is insufficient to attenuate nephrotoxicity [69]. In addition, antioxidants, such as vitamin E, vitamin C, selenium, carotenoids, melatonin, edaravone, and N-acetylcysteine, have been reported to reduce the nephrotoxicity of CIS, but have only been evaluated in animal models [70,71,72,73,74,75,76]. On the other hand, an organic cation transporter 2 inhibitor, cimetidine, that blocks CIS transport to the kidneys, carvedilol, which inhibits oxidative stress, cilastatin, which blocks the apoptotic pathway, rosiglitazone, which reduces inflammation, and amifostine, which exerts cytoprotective effects, have been applied to reduce the nephrotoxicity of CIS [18,77,78,79,80,81]. However, not only are these drugs less effective in reducing side effects, but they themselves cause side effects. Furthermore, recent in vitro and in vivo studies demonstrated that natural products, such as flavonoids, saponins, alkaloids, polysaccharides, and phenylpropanoids, inhibit oxidative stress, inflammation, and apoptosis in CIS-induced acute kidney injury [18,82,83,84,85,86]. Further development, including clinical studies, is awaited.

### 4.2. Cardiotoxicity of DXR

Various therapeutic substances have been developed to reduce the cardiotoxicity of DXR. Dexrazoxane is an iron chelator that reduces cardiotoxicity by inhibiting the formation of the iron–DXR complex, thereby decreasing ROS [87]. This drug was approved by the U.S. Food and Drug Administration (FDA) as a drug that reduces chemotherapeutic agent-induced cardiotoxicity, including DXR [88]. However, subsequent clinical studies raised questions about its efficacy, and there are also concerns about the possibility of secondary carcinogenesis induced by the drug [89]. On the other hand, dexrazoxane also inhibits the formation of DNA-topoisomerase complexes by anthracyclines and is marketed as a treatment for the extravascular leakage of these anticancer drugs [90]. Other compounds, such as digoxin, which reduces oxidative stress and cell damage, autophagy-related (ATG) 7 activator, resveratrol, which reduces ROS and activates ATG7, and herbal compounds with antioxidant properties have been shown to protect against DXR-induced cardiotoxicity; however, all have efficacy and safety concerns [19,91,92,93,94]. In addition, recent molecular mechanism studies on anthracyclines revealed that DXR inhibits heme synthesis and intercalates mitochondrial DNA (mtDNA), thereby inducing ferroptosis, iron-dependent cell death, leading to cardiotoxicity [95]. An amino acid, 5-aminolevulinic acid (5-ALA), has been reported to reduce DXR-induced cardiotoxicity in mice by inhibiting the molecular mechanism responsible, and future clinical studies on 5-ALA as a protective agent against DXR-induced cardiotoxicity are anticipated [96].

## 5. Reduction in Side Effects of Anticancer Drugs by H_2_

Our literature review revealed that H_2_ reduced the side effects of CIS-induced nephrotoxicity, ototoxicity, and ovarian injury, CIS- or OXA-induced peripheral neuropathy, DXR-induced cardiotoxicity and hepatotoxicity, and BLM- or GEF-induced lung injury. A drug-specific summary of the reduction in these side effects by H_2_ is shown in Table 1 [31,32,33,34,35,36,37,38,39,40,41,42,43,44].

### 5.1. Effects on CIS- and OXA-Induced Toxicity

#### 5.1.1. Nephrotoxicity

Nakashima-Kamimura et al. investigated the mitigating effects of H_2_ gas and H_2_-rich water (HRW) on CIS-induced nephrotoxicity in mice and demonstrated that the inhalation of H_2_ gas or drinking HRW improved mortality and weight loss caused by CIS, as well as increased serum creatinine and blood urea nitrogen (BUN) levels [31]. In addition, a histopathological examination of the attenuating effects of HRW on nephrotoxicity showed that HRW decreased the number of terminally deoxynucleotidyl transferase-mediated biotinylated UTP nick end-labeling (TUNEL)-positive cells [31]. Furthermore, H_2_ did not impair the anticancer activity of CIS in in vitro experiments with cancer cell lines or in in vivo experiments with carcinoma-bearing mice [31]. These findings indicate that H_2_ alleviates CIS-induced nephrotoxicity by reducing oxidative stress and apoptosis without impairing the anticancer effects of CIS. Kitamura et al. also investigated the effects of HRW on CIS-induced nephrotoxicity in rats using dynamic contrast-enhanced computed tomography (DCE-CT) and showed that HRW mitigated CIS-induced decreases in contrast clearance per unit renal volume (K1) and contrast clearance from the entire kidney, which is K1 multiplied by the kidney volume [32]. Moreover, it reduced CIS-induced increases in serum creatinine levels and histopathological damage [32]. Collectively, these findings confirmed the protective effects of H_2_ against nephrotoxicity using DCE-CT. Matsushita et al. used blood oxygenation level-dependent (BOLD) magnetic resonance imaging (MRI) to elucidate the mechanisms responsible for the protective effects of HRW against CIS-induced nephrotoxicity in rats [33]. They demonstrated that HRW significantly ameliorated CIS-induced decreases in the parent transverse relaxation rate and increases in serum creatinine and BUN levels [33]. These findings confirmed the reno-protective effects of H_2_ using BOLD-MRI.

#### 5.1.2. Ototoxicity

Qu et al. examined the protective effects of H_2_ gas against CIS-induced ototoxicity in rats [34]. They found that the inhalation of H_2_ gas significantly improved the auditory brainstem response (ABR) and histopathologically confirmed hair cell damage. The inhalation of H_2_ gas also significantly reduced CIS-induced increases in malondialdehyde (MDA) and 8-iso-prostaglandin F2α (8-iso-PGF2α) levels in serum and cochlear tissue [34]. These findings suggest that H_2_ ameliorated CIS-induced ototoxicity by reducing oxidative stress. Kikkawa et al. also investigated the protective effects of H_2_-containing culture medium on a cultured mouse cochlear explant and reported that H_2_ significantly ameliorated CIS-induced reductions in the number of hair cells and significantly reduced the fluorescence intensity of •OH in the spiral ganglia [35]. These findings indicate that H_2_ attenuated CIS ototoxicity by reducing ROS-induced oxidative stress. Furthermore, Fransson et al. investigated the protective effects of H_2_ gas against CIS-induced ototoxicity in guinea pigs and reported that H_2_ gas mitigated CIS-induced shifts in the ABR threshold, the loss of hair cells, and reduced synaptophysin immunoreactivity [36]. They also demonstrated that H_2_ gas significantly ameliorated CIS-induced reductions in the intensity of copper transporter 1 in inner hair cells and synaptic areas around vascular strips [36]. These findings suggest that H_2_ protected against CIS-induced ototoxicity at the functional, cellular, and intracellular levels.

#### 5.1.3. Ovarian Injury

Meng et al. investigated the protective effects of H_2_-rich saline (HRS) against CIS-induced ovarian injury in rats [37]. They found that an intraperitoneal injection of HRS significantly ameliorated CIS-induced decreases in estrogen and increases in follicle-stimulating hormone in ovarian tissue as well as histopathologically confirmed ovarian cortical injury [37]. HRS also significantly attenuated CIS-induced decreases in superoxide dismutase (SOD) and catalase (CAT) and increases in MDA. In addition, HRS enhanced the CIS-induced increase in nuclear factor erythroid 2-related factor 2 (Nrf2) expression in ovarian tissue [37]. These findings indicate that H_2_ protected against CIS-induced ovarian injury by regulating oxidative stress, which, in turn, involved the activation of Nrf2.

#### 5.1.4. Peripheral Neuropathy

Martínez-Martel et al. examined the preventive effects of HRW against allodynia and functional and emotional deficits induced by CIS in female and male mice [38]. The findings obtained demonstrated that HRW ameliorated CIS-induced allodynia and functional and emotional deficits [38]. HRW also significantly attenuated CIS-induced increases in NLRP3 and 4-hydroxy-2-nonenal (4-HNE) protein levels in the dorsal root ganglia and prefrontal cortex and decreases in the expression of heme oxygenase-1 (HO-1) and SOD [38]. These findings indicate that H_2_ protected against CIS-induced allodynia and functional and emotional deficits by reducing oxidative stress and inflammation. Lian et al. examined the effects of HRW on OXA-induced neuropathic pain in mice and demonstrated that HRW alleviated hyperalgesia induced by OXA in mice, decreased the diversity of intestinal bacteria, and changed the structure of the intestinal microbiota [39]. HRW also reversed the imbalance of inflammatory cytokines, such as tumor necrosis factor (TNF)-α and IL-6, and oxidative stress factors, including •OH and ONOO^−^, in cord segments and decreased the expression of lipopolysaccharide (LPS) and toll-like receptor 4 (TLR4) [39]. These findings indicate that H_2_ mitigated neuropathic pain through changes in the diversity and structure of the gut microbiota as well as the LPS-TLR4 pathway.

### 5.2. Effects on DXR-Induced Cardiotoxicity and Hepatotoxicity

Gao et al. examined the effects of an intraperitoneal injection of HRS on DXR-induced cardiotoxicity and hepatotoxicity in rats [40]. The findings obtained showed that HRS ameliorated DXR-induced mortality, cardiac dysfunction, and histopathological injury in the heart and liver of rats. HRS also significantly attenuated DXR-induced increases in serum brain natriuretic peptide, aspartate transaminase, and alanine transaminase as well as ROS and MDA in the heart and liver [40]. In addition, HRS significantly attenuated DXR-induced increases in the inflammation-related markers TNF-α, IL-1β, and IL-6 in the heart and liver as well as the apoptosis-related markers TUNEL, Bcl-2-associated x (Bax), and the B-cell/CLL lymphoma 2 (Bcl-2) ratio, and significantly reduced increases in caspase-3 and caspase-8 levels [40]. These findings indicate that H_2_ ameliorated DXR-induced cardiotoxicity and hepatotoxicity by attenuating inflammation and apoptosis.

Ma et al. examined the effects of H_2_ gas inhalation in a rat model of DXR-induced myocardial injury and demonstrated improvements in cardiac function and the attenuation of histopathological damage to the myocardium [41]. H_2_ gas also restored DXR-induced decreases in the expression of microtubule-associated protein light chain 3 (LC3), an autophagy-related protein, in cardiomyocytes and simultaneously ameliorated DXR-induced increases in apoptosis (TUNEL, Bax/Bcl-2 ratio, caspase-3, and caspase-9) [41]. Furthermore, H_2_ gas increased the ratio of phosphorylated AMP-dependent protein kinase (p-AMPK) to AMPK and decreased the phosphorylated mammalian target of rapamycin (p-mTOR) to mTOR ratio [41]. These findings indicate that H_2_ activated autophagy via the AMPK/mTOR pathway and protected against DXR-induced myocardial injury.

### 5.3. Effects on BLM-Induced Lung Injury

Gao et al. examined the effects of H_2_ gas inhalation in a rat model of BLM-induced lung fibrosis and found that it reduced BLM-induced increases in ROS, MDA, transforming growth factor (TGF)-β1, and TNF-α levels in lung tissue and attenuated BLM-induced reductions in the activity of glutathione peroxidase (GSH-PX). H_2_ gas also inhibited BLM-mediated epithelial-to-mesenchymal transition by increasing the expression level of the epithelial cell marker E-cadherin and decreasing that of the mesenchymal cell marker vimentin [42]. Furthermore, H_2_ gas exerted its antifibrotic effect by down-regulating the expression of α-smooth muscle actin and inhibiting collagen I production [42]. These findings indicate that H_2_ alleviated BLM-induced pulmonary fibrosis by suppressing fibrosis-related TGF-β1 and oxidative stress as well as inhibiting epithelial-to-mesenchymal transition. Aokage et al. investigated the effects of H_2_ gas inhalation in a mouse model of BLM-induced pulmonary fibrosis and found that H_2_ gas reduced the BLM-induced deterioration of respiratory function and histopathologically confirmed lung fibrosis [43]. They also showed that H_2_ gas suppressed increases in IL-6, IL-4, and IL-13 levels in alveolar macrophages, decreased the number of M2-biased macrophages that function in lung fibrosis, and reduced the number of TGF-β1-secreting cells that induce fibrosis [43]. These findings indicate H_2_ ameliorated BLM-induced respiratory dysfunction and pulmonary fibrosis.

### 5.4. Effects on GEF-Induced Lung Injury

Terasaki et al. examined the effects of HRW in a mouse model of acute lung injury induced by the post-administration of GEF after the pre-administration of naphthalene, a toxic agent associated with oxidative stress, and reported that HRW reversed weight loss, prolonged survival, ameliorated lung histopathological changes, and increased cell counts in bronchoalveolar lavage fluid in mice treated with naphthalene/GEF [44]. However, H_2_ attenuated naphthalene-induced decreases in GSH levels and increases in MDA and 4-HNE levels, and exerted antioxidant effects, but did not significantly affect naphthalene/GEF-induced changes [44]. On the other hand, H_2_ did not impair the anticancer effects of GEF in in vitro experiments on lung cancer cell lines or in in vivo experiments on carcinoma-bearing mice [44]. These findings indicate that H_2_ reduced GEF-induced lung injury without impairing its anticancer activity.

## 6. Mechanisms by Which H_2_ Reduces Side Effects of Anticancer Drugs

**Figure 2:**
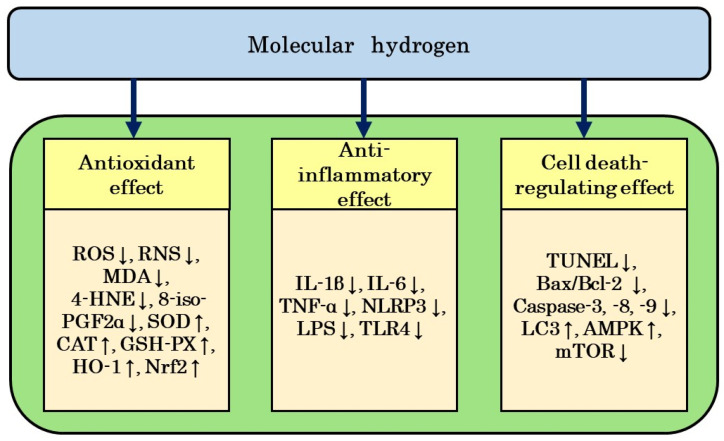
Possible mechanisms underlying protective effects of H_2_ against tissue injuries induced by anticancer drugs. Protective effects are mainly categorized as antioxidant, anti-inflammatory, and cell death-regulating effects. AMPK: AMP-dependent protein kinase; Bcl-2: B-cell/CLL lymphoma 2; Bax: Bcl-2-associated x; CAT: catalase; GSH-PX: glutathione peroxidase; H_2_: molecular hydrogen; 4-HNE: 4-hydroxy-2-nonenal; HO-1: heme oxygenase-1; IL: interleukin; 8-iso-PGF2α: 8-iso-prostaglandin F2α; LPS: lipopolysaccharide; LC3: microtubule-associated protein light chain 3; MDA: malondialdehyde; mTOR: mammalian target of rapamycin; Nrf2: nuclear factor erythroid 2-related factor 2; NLRP3: NLR family pyrin domain containing 3; RNS: reactive nitrogen species; ROS: reactive oxygen species; SOD: superoxide dismutase; TNF-α: tumor necrosis factor-α; TLR4: Toll-like receptor 4; TGF-β1: transforming growth factor-β1; TUNEL: terminally deoxynucleotidyl transferase-mediated biotinylated UTP nick end-labeling; ↑: increase; ↓: decrease.

Our literature review revealed that H_2_ reduced the side effects of CIS, OXA, DXR, BLM, and GEF, mainly due to its antioxidant and anti-inflammatory effects, but also through its anti-apoptotic and autophagy-activating effects, as well as its cell death-regulating effects. These effects do not exist in isolation but appear to affect each other and constitute the overall capacity of H_2_ to reduce side effects. These effects are described for each item (Figure 2).

### 6.1. Antioxidant Effects

In studies that examined the efficacy of H_2_ against CIS-induced auditory toxicity [34], OXA-induced peripheral neuropathy [39], DXR-induced cardiotoxicity and hepatotoxicity [40], and BLM-induced lung injury [42], H_2_ reduced the production of ROS and RNS, i.e., •OH and ONOO^−^, induced by the side effects of these anticancer drugs. On the other hand, in studies that investigated the efficacy of H_2_ against CIS-induced ototoxicity [34], ovarian injury [37], and peripheral neuropathy [38], DXR-induced cardiotoxicity and hepatotoxicity [40], and BLM-induced lung injury [42], H_2_ reduced MDA, 8-iso-PGF2α, and 4-HNE levels, which were increased by the side effects of these anticancer drugs, and also enhanced the activities of SOD, CAT, HO-1, and GSH-PX, which were decreased by these anticancer drugs.

These findings may be attributed to H_2_ not only directly inhibiting ROS production, but also indirectly regulating enzymes involved in oxidation and anti-oxidation, resulting in antioxidant effects. ROS are involved in the mechanisms responsible for the cytotoxic effects of CIS, DXR, and BLM in cancer cells. However, from the perspective of reducing side effects, it is important to suppress the production of ROS, which cause tissue damage, and prevent them from causing secondary damage.

### 6.2. Anti-Inflammatory Effects

In studies that examined the efficacy of H_2_ against peripheral neuropathy induced by CIS and OXA [38,39], cardiotoxicity and hepatotoxicity induced by DXR [40], and lung injury induced by BLM [42,43], H_2_ reduced NLRP3 protein levels, LPS and TLR4 expression levels, and TNF-α, IL-1β, and IL-6 levels, respectively, which were increased by these anticancer drugs. These findings suggest the involvement of anti-inflammatory effects in the H_2_-induced attenuation of the side effects of anticancer drugs.

H_2_ also demonstrated efficacy against GEF-induced lung injury; however, the underlying mechanisms remain unclear. The mechanisms by which GEF induces lung injury involve its induction of macrophages to activate NLRP3 and release IL-1β as well as the release of HMGB1 via the abnormal activation of PARP-1 [67]. Therefore, in GEF-induced lung injury, H_2_ may reduce lung injury by blocking the pathway leading to the release of inflammatory cytokines via the activation of NLRP3. This is supported by our previous review showing that the inhibition of mtDNA oxidation by H_2_ may inhibit the cascade from NLRP3 activation to the release of inflammatory cytokines, thereby suppressing chronic inflammation [97].

### 6.3. Regulation of Cell Death

In studies that investigated the efficacy of H_2_ against CIS-induced nephrotoxicity [31] and DXR-induced cardiotoxicity and hepatotoxicity [40,41], H_2_ reduced the increase in or elevated TUNEL-positive cell numbers and TUNEL protein levels, the Bax/Bcl-2 ratio, and caspase-3, -8, and -9 activities, respectively. These findings suggest that H_2_ reduced the toxicity of CIS and DXR through its anti-apoptotic effects. On the other hand, in a study on DXR-induced cardiotoxicity [41], H_2_ restored DXR-induced decreases in the expression of LC3, increased the p-AMPK to AMPK ratio, and reduced the p-mTOR to mTOR ratio. These findings indicate that H_2_ activated autophagy through the AMPK/mTOR pathway and protected against DXR-induced cardiotoxicity. These findings also suggest the involvement of cell death-regulating effects, such as the inhibition of apoptosis and activation of autophagy, in the mechanisms by which H_2_ mitigates the toxic effects of CIS and DXR.

## 7. Anticancer and Radioprotective Effects of H_2_

### 7.1. Anticancer Effects

The anticancer effects of H_2_ have been reported in many in vitro and in vivo studies, and clinical trials recently demonstrated that inhalation therapy with H_2_ gas was effective against various cancers [27]. Chen et al. showed that H_2_ gas inhalation therapy for 82 cancer patients over 3 months improved their quality of life and suppressed cancer progression [30]. They used H_2_ gas inhalation in combination with several small-dose anticancer drugs to treat approximately 2/3 of their patients but did not observe any significant difference in anticancer effects between H_2_ gas alone and its combination with drugs or any reduction in side effects when drugs were used together [30]. Akagi et al. examined the effects of the combination of the anti-PD-1 antibody nivolumab and H_2_ gas inhalation in cancer patients for ≥60 months and reported that 42 patients treated with the combination achieved a significant overall survival advantage in contrast to 14 patients treated with nivolumab alone [45].

Chen et al. divided 58 patients with non-small cell lung cancer into five groups: a control group (10 patients), H_2_ alone group (10 patients), H_2_ plus chemotherapy group (10 patients), H_2_ plus targeted therapy group (18 patients), and H_2_ plus immunotherapy group (10 patients) [46]. The combination therapy groups received chemotherapy drugs, including CIS or carboplatin, several targeted therapy drugs, including GEF, and immunotherapy drugs, including nivolumab or pembrolizumab. The H_2_ alone and H_2_ combination groups, excluding the control group, received H_2_ gas inhalation for 5 months. During the first 5 months of treatment, the prevalence of symptoms gradually increased in the control group and gradually decreased in the four treatment groups [46]. In a 16-month follow-up, the H_2_ alone, H_2_ plus chemotherapy, H_2_ plus targeted therapy, and H_2_ plus immunotherapy groups had significantly higher progression-free rates than the control group [46]. The side effects of most anticancer drugs were also reduced or eliminated in the combination therapy groups [46]. These findings indicate that the inhalation of H_2_ gas inhibited tumor progression in non-small cell lung cancer patients. Although the side effect-reducing effects of individual anticancer drugs were not described and H_2_ was clearly shown to alleviate the various side effects of chemotherapy drugs, targeted therapy drugs, and immunotherapy drugs, the findings obtained indicate that the combination of H_2_ and anticancer effects was generally effective in reducing the side effects of anticancer drugs.

**Figure 3:**
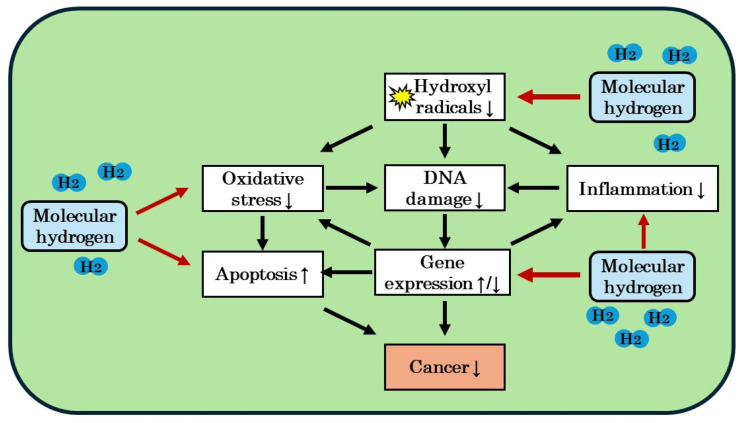
Possible mechanism of the anticancer effects of molecular hydrogen (H_2_). H_2_ reduces hydroxyl radicals directly, and also exhibits antioxidant, anti-inflammatory, and apoptotic effects via the regulation of gene expression indirectly. Through these effects, H_2_ may exhibit anticancer effects. See [27] for the details.↑: increase; ↓: decrease; ↑/↓: increase or decrease.

Therefore, the combination of H_2_ and anticancer drugs may not only enhance the anticancer effects of anticancer drugs, but also alleviate their side effects [27,30,45,46]. The finding showing that H_2_ enhanced the anticancer effects of anticancer drugs without impairing their anticancer effects suggests that an indirect defense mechanism through the regulation of gene expression is more closely involved in the anticancer effects of H_2_ than the direct reduction of •OH (Figure 3) [27].

### 7.2. Radioprotective Effects

The mechanisms of injury inflicted on the organism by anticancer drugs and radiation are very similar. We have reported the protective effect of H_2_ against radiation injury and its possible mechanisms in a previous review [25]. In this chapter, we compare the mechanisms by which H_2_ exerts protective effects against anticancer drug-induced injury with those by which it exerts protective effects against radiation-induced injury.

The harmful effects of ionizing radiation on the body are classified into direct and indirect effects. Direct effects are those caused by the direct absorption of radiation energy into nucleic acids (DNA), proteins, and lipids [98,99,100,101]. Indirect effects are those caused by free radicals, such as •OH, and molecules produced in the process of water radiolysis [98,99,100,101]. Since low-dose radiation damage is mainly caused by this indirect effect, secondary damage as a non-DNA target effect is more closely involved than direct damage to DNA [98,99,100,101]. This secondary damage includes oxidative stress associated with intracellular responses, inflammation, apoptosis, and effects on gene expression.

**Figure 4:**
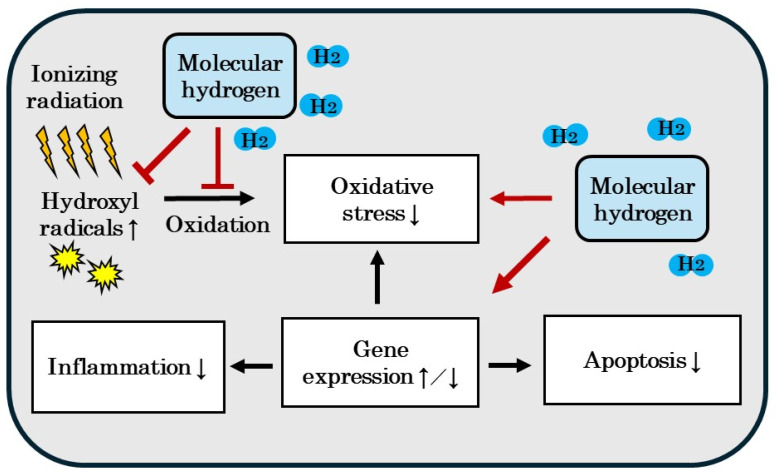
Molecular hydrogen (H_2_) not only has a direct radioprotective effect by reducing hydroxyl radicals, but also indirectly by regulating by gene expression, exhibiting antioxidant, anti-inflammatory and anti-apoptotic effects, which may lead to radioprotective effects. See [25] for the details. ↑: increase; ↓: decrease; ↑/↓: increase or decrease.

The radioprotective effects of H_2_ have also been reported in many in vitro and in vivo studies, and clinical trials recently showed that the inhalation of H_2_ gas mitigated decreases in quality of life and bone marrow damage associated with radiation [25,102,103]. In our previous review, we described in vitro and in vivo studies in which the mechanisms by which H_2_ exerts radioprotective effects and anticancer effects were not only attributed to the direct reduction of •OH by H_2_, but also the indirect regulation of gene expression by H_2_ via intracellular responses and indirect antioxidant and anti-inflammatory effects [25]. The possibility that H_2_ may exert antioxidant, anti-inflammatory, and anti-apoptotic effects indirectly by regulating gene expression via intracellular responses was demonstrated (Figure 4) [25]. Therefore, the mechanisms by which H_2_ exerts protective effects against radiation- and anticancer drug-induced cytotoxicity are similar.

## 8. Prospects for the Clinical Application of H_2_

This literature review demonstrated that H_2_ reduced CIS-induced nephrotoxicity, ototoxicity, and ovarian injury, CIS- or OXA-induced peripheral neuropathy, DXR-induced cardiotoxicity and hepatotoxicity, and BLM- or GEF-induced lung injury [26,27,28,29,30,31,32,33,34,35,36,37,38,39]. Analyses of the mechanisms by which H_2_ reduces side effects based on changes in biochemical and molecular biological markers revealed that the antioxidant, anti-inflammatory, and cell death-regulating effects of H_2_ were protective against CIS- and DXR-induced disorders [31,32,33,34,35,36,37,38,40,41]. Furthermore, the antioxidant and anti-inflammatory effects of H_2_ appeared to be protective against OXA-, BLM-, and GEF-induced disorders [39,42,43,44]. These findings allow us to conclude that H_2_ mainly protected against various tissue injuries from the side effects of anticancer drugs in animal studies by reducing oxidative stress and inflammation.

A number of efforts have been made to reduce the side effects of anticancer drugs. Research on the development of derivatives and analogues of existing anticancer drugs with fewer side effects and research on formulation technologies, such as liposomes and emulsions, have been conducted without satisfactory outcomes [14,15,16,17]. The mechanisms of nephrotoxicity by CIS and cardiotoxicity by DOX involve oxidative stress, inflammation, and apoptosis. Therefore, antioxidant, anti-inflammatory, and anti-apoptotic substances have been used in combination, and some have been applied to clinical trials [18,19]. However, satisfactory findings have yet to be reported.

The attenuating effects of H_2_ on the side effects of anticancer drugs have mainly been examined in animal studies, and the underlying mechanisms have been attributed to its antioxidant, anti-inflammatory, anti-apoptotic, and autophagy-activating properties [31,32,33,34,35,36,37,38,39,40,41,42,43,44]. Clinical studies confirmed the attenuating effects of H_2_ on the side effects of anticancer drugs; however, they were not detailed clinical studies specific to side effect reductions [46]. Furthermore, in animal studies, the combination of H_2_ and anticancer drugs did not impair the anticancer effects of anticancer drugs [31,44], and clinical studies demonstrated that the combination of H_2_ gas and anticancer drugs enhanced the anticancer effects of anticancer drugs more than each alone [45]. The efficacy of H_2_ in the treatment of various human diseases has been reported in more than 150 clinical studies, and no adverse events have been attributed to H_2_. Therefore, the combination of H_2_ and anticancer drugs for cancer patients may be a safe and effective treatment method that enhances anticancer effects and reduces side effects.

On the other hand, there are a number of limitations that need to be addressed. Only five anticancer drugs, CIS, OXA, DXR, BLM, and GEF, were discussed in this review. Although it is likely that H_2_ exerts similar effects on the side effects of many anticancer drugs other than those discussed herein, further studies are needed to expand the range of anticancer drugs. Furthermore, H_2_ has been shown to ameliorate tissue damage as a side effect of anticancer drugs by reducing oxidative stress and inflammation; however, the underlying molecular mechanisms, including the target molecules responsible for these effects, remain unknown. Recent studies reported that the in vivo reduction of •OH by H_2_ requires the oxidation of iron–porphyrin as a catalyst, and that this iron–porphyrin oxide is the target molecule of H_2_ [22]. However, the molecular mechanisms underlying the efficacy and attenuation of side effects, including the target molecule of H_2_, are still unclear and require further study. In addition, recent studies have reported that biopolymers have promising potential as a new class of materials for use in biomedical applications [104,105]. Hence, the potential of integrating biopolymers with H_2_ to augment anticancer efficacy or mitigate the side effects of anticancer drugs presents an intriguing avenue for future exploration.

## 9. Conclusions

While H_2_ has been reported to mitigate the side effects of anticancer drugs in animal studies and clinical trials, our understanding of its molecular mechanisms remains limited. To address this, we conducted an extensive review of animal studies. The findings obtained indicate that H_2_ protects against nephrotoxicity, ototoxicity, and ovarian injury induced by CIS, peripheral neuropathy caused by CIS or OXY, cardiotoxicity and hepatotoxicity resulting from DXR, and lung injury due to BLM or GEF. The primary mechanisms involved appear to be the reduction in oxidative stress and inflammation. In clinical studies, H_2_ exhibited efficacy in reducing the side effects of anticancer drugs. Interestingly, H_2_ did not diminish the anticancer effects of these drugs in animal experiments. The combination of H_2_ and anticancer drugs enhanced anticancer effects in clinical trials. Therefore, the combination of H_2_ and anticancer drugs has potential as a treatment strategy that reduces the side effects of anticancer drugs and enhances their anticancer effects. This presents an exciting avenue for future cancer treatments.

## Figures and Tables

**Table 1:** Summary of protective effects of H_2_ against various tissue injuries induced by anticancer drugs in animal models.

Anticancer Drugs	Toxicities/Injuries	Changes in Biomarkers	Ref. No.
CIS	Nephrotoxicity	BUN ↓, Creatinine ↓, TUNEL ↓	[31]
		Creatinine ↓	[32]
		BUN ↓, Creatinine ↓	[33]
CIS	Ototoxicity	MDA ↓, 8-iso-PGF2α ↓	[34]
		•OH ↓	[35]
		Synaptophysin ↑, Copper transporter 1 ↑	[36]
CIS	Ovarian injury	SOD ↑, CAT ↑, MDA ↓, Nrf2 ↑	[37]
CIS	Peripheral neuropathy	NLRP3 ↓, 4-HNE ↓, HO-1 ↑, SOD ↑	[38]
OXA	Peripheral neuropathy	TNF-α ↓, IL-6 ↓, •OH ↓, ONOO^−^ ↓, LPS ↓, TLR4 ↓	[39]
DXR	Cardiotoxicity and hepatotoxicity	BNP ↓, AST ↓, ALT ↓, ROS ↓, MDA ↓, TNF-α ↓, IL-1β ↓, IL-6 ↓, TUNEL ↓, Bax/Bcl-2 ↓, Caspase-3 ↓, Caspase-8 ↓	[40]
DXR	Cardiotoxicity	LC3 ↑, TUNEL ↓, Bax/Bcl-2 ↓, Caspase-3 ↓, Caspase-9 ↓, AMPK ↑, mTOR ↓	[41]
BLM	Lung injury	ROS ↓, MDA ↓ TGF-β1 ↓,TNF-α ↓, GSH-PX ↑, E-cadherin ↑, Vimentin ↓, α-SMA ↓, Collagen I ↓	[42]
		IL-4 ↓, IL-6 ↓, IL-13 ↓ TGF-β1 ↓	[43]
GEF	Lung injury	(GSH ↓, MDA ↓, 4-HNE ↓) *	[44]

AMPK: AMP-dependent protein kinase; ALT: alanine transaminase; AST: aspartate transaminase; Bcl-2: B-cell/CLL lymphoma 2; Bax: Bcl-2-associated x; BUN: blood urea nitrogen; BNP: brain natriuretic peptide; BLM: bleomycin; CIS: cisplatin; CAT: catalase; DXR: doxorubicin; GSH: glutathione; GSH-PX: glutathione peroxidase; GEF: gefitinib; H_2_: molecular hydrogen; 4-HNE: 4-hydroxy-2-nonenal; HO-1: heme oxygenase-1; IL: interleukin; 8-iso-PGF2α: 8-iso-prostaglandin F2α; LPS: lipopolysaccharide; LC3: microtubule-associated protein light chain 3; MDA: malondialdehyde; mTOR: mammalian target of rapamycin; Nrf2: nuclear factor erythroid 2-related factor 2; NLRP3: NLR family pyrin domain containing 3; OXA: oxaliplatin; •OH: hydroxyl radicals; ONOO^−^: peroxynitrite_;_ ROS: reactive oxygen species; SOD: superoxide dismutase; α-SMA: α-smooth muscle actin; TNF-α: tumor necrosis factor-α; TLR4: Toll-like receptor 4; TGF-β1: transforming growth factor-β1; TUNEL: terminally deoxynucleotidyl transferase-mediated biotinylated UTP nick end-labeling; *: changes with naphthalene only; ↑: increase; ↓: decrease.
